# A Multi-Directional and Agile Academic Knowledge Transfer Strategy for Healthcare Technology

**DOI:** 10.3389/frobt.2021.789827

**Published:** 2021-12-21

**Authors:** Isabel Klemme, Birte Richter, Kevin De Sabbata, Britta Wrede, Anna-Lisa Vollmer

**Affiliations:** ^1^ Athena Institute, Faculty of Science, Vrije Universiteit Amsterdam, Amsterdam, Netherlands; ^2^ Medical Assistance Systems, Medical School OWL, Center for Cognitive Interaction Technology, Bielefeld University, Bielefeld, Germany

**Keywords:** knowledge transfer, technology transfer, transfer strategy, cross-sector collaboration, cognitive interaction technology, cooperation projects

## Abstract

Technology, especially cognitive agents and robots, has significant potential to improve the healthcare system and patient care. However, innovation within academia seldomly finds its way into practice. At least in Germany, there is still a digitalization gap between academia and healthcare practice and little understanding of how healthcare facilities can successfully purchase, implement, and adopt new knowledge and technology. Therefore, the aim of this study is to develop a successful academic knowledge transfer strategy for healthcare technology. We conducted a qualitative study with academic staff working in higher education in Germany and professionals in their practice partner organizations. In 15 semi-structured interviews, we aimed to assess interviewees experiences with knowledge transfer, to identify perceived influencing factors, and to understand the key aspects of a successful knowledge transfer strategy. The Dynamic Knowledge Transfer Model by Wehn and Montalvo, 2018 was used for data analysis. Based on our findings, we suggest that a successful transfer strategy between academia and practice needs to be multi-directional and agile. Moreover, partners within the transfer need to be on equal terms about expected knowledge transfer project outcomes. Our proposed measures focus particularly on regular consultations and communication during and after the project proposal phase.

## Introduction

“Knowledge transfer” (KT) refers to the process of exchanging and applying research results generated within academia in practice (Ward et al., 2009). This study focuses on technological KT which refers to the “process of conveying results from scientific and technological research to the marketplace and to society and is as such an essential part of the technological innovation process” (European Commission, 2019). There are numerous advantages resulting from the relation between research institutes and healthcare practice such as improved surgical outcomes, treatment processes and patient care (e.g., personalized treatment and tele-therapy applications) (Anderson, 2001; Collins et al., 2016). For providing patients with the best possible care, collaboration between research institutes and practice in healthcare is a key element (Collins et al., 2016). According to (Collins et al., 2016) the current challenges faced by the healthcare system are in particular the improvement of access to healthcare, the reduction of healthcare costs, and the improvement of quality of care. These three challenges can be addressed when knowledge transfer into healthcare is successful. A growing body of literature recognizes the importance of successful interdisciplinary KT, particularly in the healthcare sector (Ward et al., 2009). Further, the World Health Organization claimed that a loose working relationship between knowledge providers and recipients would ensure that research knowledge is used to improve health (World Health Organization, 2004). Moreover, the final report of the Knowledge Transfer Study 2010-2012 from the European Commission stated that KT policy is an important issue in Europe (European Commission, 2013). Therefore, the EU Framework Programme for Research and Innovation “Horizon 2020” supported science and knowledge transfer from academia to practice with almost 80 billion euros some invested in the healthcare sector. These efforts show the importance attached to KT. However, transfer activities are structurally difficult to integrate into the (German) university research context. 4 years ago, the German “Wissenschaftsrat” published a memorandum calling for more appreciation of transfer but also warned that transfer requires additional resources which should not compete with resources for teaching and research (Wissenschaftsrat, 2019).

To give recommendations for a KT strategy, the context of a research institute and the experiences made with KT by practice in healthcare and academic researchers need to be considered. Therefore, the aim of this study is to investigate challenges encountered during KT and to give recommendations to research institutes on relevant measures for a successful knowledge transfer strategy between academia and the healthcare sector. The research question was raised by a working group of a research facility in Bielefeld, Germany. This research facility focuses on cognitive sciences and interaction technology (i.e., technical systems with cognitive abilities that are able to interact naturally with people and adjust to changing situations). Even though the cutting-edge technology developed has great potential for application in the social healthcare domain, the technology readiness level of the developed systems such as cognitive humanoid robots is particularly low, due to the complexity of cognitive interaction technology. Additionally, in Germany, a large gap exists between the complex technological systems in academia and the level of digitization in the healthcare sector. However, the demand for healthcare technology and assistance systems and collaboration between academia and the healthcare sector is uniquely strong in Bielefeld because Europe’s biggest social enterprise, the “von Bodelschwinghsche Stiftungen Bethel”, is in Bielefeld. Thus, this setting is especially interesting for investigating the important challenges of current KT and to define general measures for alleviating them.

Some authors of this article were part of the KT relevant projects we considered but did not participate in the interviews. Data was collected from KT projects in the context of the research institute. While the recommendations are based on experiences from projects in a specific environment (i.e., Bielefeld, Germany), the raised issues point to more general issues which are discussed later. By analyzing the experiences both academic partners and practice partners made during KT in collaboration projects, the aim of the research was to develop a successful academic knowledge transfer strategy for healthcare technology.

## Challenges in Current KT

The main barriers to KT of technology according to Riege (2005) are inadequate technical conditions for the deployment of technological innovations and insufficient financial resources. Both of these barriers complicate work routines and communication within the organization. Additionally, they explain an increasing demand for more efficient and effective strategies for the transfer of knowledge, technologies and innovations from research into practice.

Robust research in KT in healthcare is limited (Pentland et al., 2011) and there is little understanding of how healthcare facilities should purchase, implement, and adopt new knowledge and technology (Schoville and Titler, 2015). Thus, the following key problem remains: understanding how knowledge and innovations become routinely transferred in everyday healthcare practice (May, 2013). KT models have been developed in many fields, yet their implementation in practice is still an issue and faces many challenges (Van de Ven and Johnson, 2006).

Previous innovation studies have indicated inconsistent findings regarding the implementation process of innovations in organizations (Schoville and Titler, 2015) such as healthcare organizations. Several factors hamper KT into practice, which are listed in the following. Innovation is often complex. Some innovations lead to changes in organizations which may temporarily disrupt routines in these organizations (Schoville and Titler, 2015). Furthermore, the constellation of partners involved in the KT process changes from project to project. Additionally, in the special case of healthcare, the practice partners (medical professionals) involved in the KT process are not always the end users of the innovation (patients). This can create issues which hamper KT because the KT process then might yield technology that is not tailored to the end users’ needs and requirements (Nieva et al., 2005). Not only in healthcare, practitioners often encounter the problem that innovations cannot be adopted in the field because the prototype which has been developed in the KT cannot be directly implemented in practice (Ferry et al., 2019). Thus, the readiness of the innovation for practical implementation plays an important role for a successful KT.

Another KT problem is the assumption that practical knowledge derives from research knowledge. Many academic researchers have learned that knowledge is created and tested by academic researchers, adopted by advisors, and practiced by practitioners (Van de Ven and Johnson, 2006). However, as Starkey and Madan, and Van de Ven and Johnson point out, academic researchers are not the only ones creating knowledge (Starkey and Madan, 2001; Van de Ven and Johnson, 2006). Practitioners discover problems and insights from their practices, just as researchers do. Researchers and practitioners create different knowledge which depends also on their corresponding context and purpose (Van de Ven and Johnson, 2006). A KT strategy should reflect this bi-directionality. Moreover, when using a KT strategy, research institutes are better able to adapt to the particular challenges of healthcare systems (Scott, 2007).

This work aims to fill in the lack of data on successful KT strategies for academic research institutes that collaborate with different partners in the healthcare sector. We investigate what comprises a successful technological KT strategy.

## Proposed Method and Procedure

### Dynamic Knowledge Transfer Model

**FIGURE 1:**
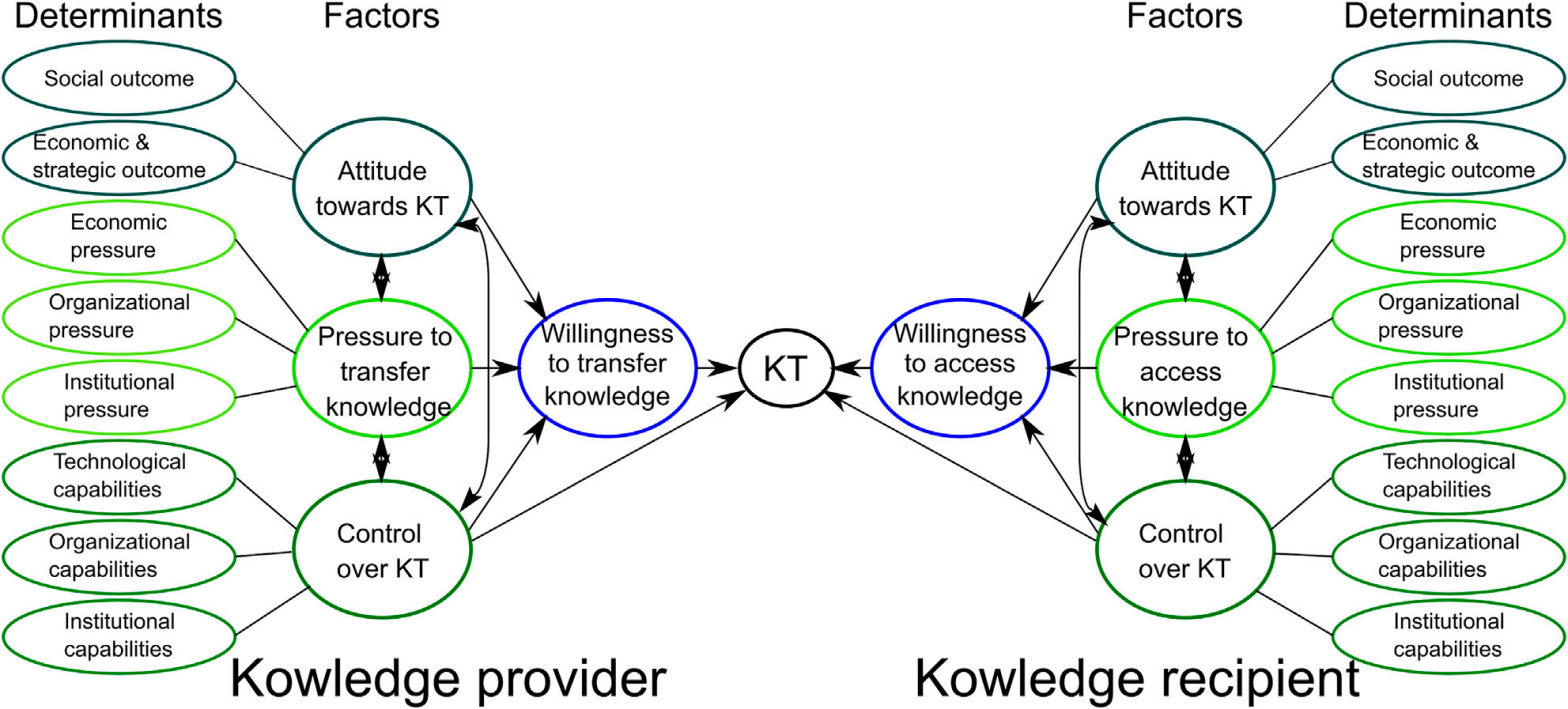
The dynamic KT model presents a variety of sources of behavioral (a)symmetries for KT. After (Wehn and Montalvo, 2018).

This study adopts a behavioral science approach, which explores the dynamic relationship between two actors. The Dynamic Knowledge Transfer Model by Wehn and Montalvo, 2018 aims to provide the basis for a new understanding of the dynamic KT across different organizations. The dynamic KT model shows three different factors which influence the dynamics of KT between provider and recipients. These are the attitude towards KT, pressure to transfer or access knowledge and the control over the KT process (Figure 1). These factors influence the willingness of the providers to transfer and recipients to access knowledge and engage in KT. Furthermore, these three factors are each determined by ambient factors (determinants) which indirectly affect the KT process.

1. Attitude of the actors towards engaging in KT is defined as evaluation or appraisal of an expected KT outcome. A positive attitude towards the expected KT outcome will positively influence the willingness to engage in KT, the opposite applies for a negative attitude towards the KT outcome (Wehn and Montalvo, 2018). Attitude towards KT is determined by desired and expected social outcomes and economic and strategic outcomes (ambient factors). For the provider, the desired social outcomes influencing the attitude could be the contribution to knowledge generation and dissemination in science. The desired economic and strategic outcomes could be for example the reception of new funding. This means that the attitude refers to specific outcomes that are valued positively or negatively. It can be expected that actors will prefer behavior that implies desirable consequences. This will influence the willingness of the provider and recipient to engage in KT.

2. Pressure to transfer or access knowledge means that the actors feel obliged to engage in the KT because of contextual social norms of organizations or institutions (Wehn and Montalvo, 2018). Pressure to transfer or access knowledge is determined by three ambient factors: organizational pressure, economic pressure and institutional pressure. For the provider, perceived organizational pressure can arise from KT being a mandate because research funding is financed by taxes. Perceived economic pressure may result from the fact that the allocation of research funds presupposes that KT takes place. Perceived institutional pressure can arise for instance from national policies that show the high importance of science based innovation in healthcare. For the recipient, perceived economic pressure can arise from customers’ or patients’ expectations or pace of technological innovation in the sector. Perceived organizational pressure can arise through staff suggestions or management expectations. The perceived institutional pressure can arise from national and local policies. Depending on the pressure perceived, the willingness to engage in KT of the provider or recipient is expected strong or weak. In general, pressure arises from the context in which the actors operate.

3. Control over KT means the perceived ease or difficulty to perform a behavior and whether the needed resources are available for the organization to carry out the transfer of knowledge (Wehn and Montalvo, 2018). Control over KT is determined by three ambient factors technological capabilities, organizational capabilities and institutional capabilities. For the provider, technological capabilities can be influenced by the provider programming already existing robots or programming robots under the aspect of possible cognitive interaction with humans. Organizational capabilities can be influenced by knowledge management, management of KT process, availability of researchers and resources, duration of research funding. The provider’s institutional capabilities can be influenced by the financial situation because of time limited funding. This will further help to determine which factors within the provider’s strategy should be changed in order to enable successful KT to their practice partners. For the recipient, technological capabilities can be influenced by absorptive capacity, knowledge of technology and interoperability with existing technology. Organizational capabilities can be influenced by knowledge management, management of the KT process, availability of staff and resources. For institutional capabilities further insights can only be established after the interviews. Once established, it can give indications of how the institutional capabilities of the recipients can influence their control over the KT process. These ambient factors function as requisite resources to carry out the KT. Overall perceived control over the KT process arises from the beliefs regarding the perceived ease or difficulty to achieve the planned outcomes stemming from KT. Depending on the perceived control over technological, organizational and institutional capabilities the willingness of the organization to engage in KT can be expected to be either strong or weak.

The model provides a basis to systematically assess the direct effects and the sources of indirect effects of the behavior of the knowledge providers and the knowledge recipients (Wehn and Montalvo, 2018). This model is able to represent the complexity of a KT project and identify key measures that facilitate KT to practice partners in healthcare.

We apply this model to the specific case of a research institute in Germany with the aim to investigate factors accounting for successful KT and to derive necessary components for a KT strategy in the health sector.

### Methods

Necessary factors that facilitate KT between research institutes and practice partners are identified in a qualitative research study considering a research institute in Germany. An interpretive design was chosen to analyze experiences of knowledge providers and recipients in the KT process as well as their perspectives, and to give recommendations to the research institute.

#### Data Collection

The study consisted of 15 semi-structured interviews with academic staff (*n =* 7 professors and postdoctoral researchers, 3 female, 4 male) in the field of intelligent systems and technology in healthcare and staff at practice partners (*n =* 8 heads of department, 2 female, 6 male) in healthcare technology and economy (a hospital, a digital innovation and engineering company and a manufacturer of household appliances) and social institutions (foundations and aid organizations for people with disabilities). We evaluated both, academia and practice partners to understand how the actors influence each other during KT projects. The chosen sampling strategy combined elements of purposive sampling and snowball sampling procedures. Purposeful sampling involved identifying and selecting individuals that are especially knowledgeable about or experienced with the phenomenon of interest, in this case KT. The sampling process was initiated by an independent expert (who was not interviewed), but exclusively served as “trigger” to recruit and connect the researcher to the first interviewee. The sampling process was continued by snowball sampling until the sample size of 15 participants was reached. Interviews were carried out on a 1:1 basis, in anonymous form with open-ended questions. Interviews were carried out *via* telephone or video conference. The interview guide, given in Supplementary Material, was designed to explore the role of different factors on KT based on the conceptual dynamic KT model. For instance, the pressure to engage in KT was elicited by asking participants whether they felt that anyone in their institution, research group, company or community expected them to engage in KT. The interviews lasted up to 1 h.

#### Data Analysis

The interviews were recorded and transcribed verbatim with the participants’ permission. Interview data were coded using a combined deductive (initial codes were generated based on the model) and inductive (additional codes were generated as required) approach to coding. The coding process of this study was facilitated through the assistance of specialized computer software for qualitative research, ATLAS. ti.

#### Data Validity, Reliability, and Saturation

The recorded interviews were immediately transcribed and translated (German-English) after the interview. Respondents were given the opportunity to add or adjust information for accuracy of representation. Several measures ensured reliability during data gathering and analysis of this study. The interview guide was constructed in a dialogue with academic staff of the research institute to minimize the risk of researcher bias. Furthermore, open questions were included in the interview guide to minimize the bias of research subjectivity. To ensure reliability, stakeholders from different KT partners who were involved in different KT projects (*Data Collection* Section) were interviewed whose different opinions can be contrasted and thus triangulation was created within the data set. To ensure data saturation, interviews were held until no new information arose.

#### Ethical Considerations

The research design for this study was based on interviews of academic and practice partners. It was reviewed and approved by the Bielefeld University Ethics Committee. Participants provided informed consent before their interview. No personal data was requested or stored.

## Results

Interview participants engaged in projects with academic research groups and some even engaged in the same KT project. However, interviewees were interviewed individually and reported their individual experiences with KT in general (i.e., not limited to a certain project). When conducting the interviews, we identified two groups of practice partners: social partners (*n* = 4, foundations and aid organizations for people with disabilities) and economic partners (*n* = 4, a hospital, a digital innovation and engineering company and a manufacturer of household appliances). We will use the terms academic partners and practice partners in the following for what has classically been referred to as providers and recipients in KT (as suggested by Wehn and Montalvo, 2018) because in this work we postulate the importance of reciprocity in a successful transfer strategy. We use the terms practice partner and more specifically economic partner and social partner to refer to the institutions involved in a KT project with the academic partner. When referring to the individuals who participated in our study, we use the term interviewee and depending on their employment we specify that they come from academia or practice partners and more specifically from social or economic partners.

Data showed that KT from science into practice is equally important for both academic partners and practice partners. All interviewees were engaged in a KT project between academia and practice in healthcare. Twelve interviewees had experience only in academia or practice, yet three interviewees from practice partners had prior project experience in the academic sector as well.

The analysis is based on the factors of the dynamic KT model (Figure 1). Regarding the determinants in the model, the attitude towards KT and the control over KT and their respective determinants were reported to have a major influence on the willingness to transfer or access knowledge and thus on the success of KT. Pressure was not perceived to be a relevant influencing factor by the interviewees and will thus not be further elaborated upon in this article. Interestingly, organizational capability was both, the most mentioned success determinant but also the determinant with which most academic partners and practice partners experienced problems in KT projects. In the following, we will report our findings for each of the model factors and their determinants.

### Attitude Towards KT

Interestingly, all interviewees named different, as well as overlapping expected KT outcomes, to positively influence their attitude towards KT. The differences in expected outcomes were found to arise from the different interests, mandates and basic conditions of the academic partners and practice partners as elaborated in the following. Interviewees shared a mixture of expected outcomes for engaging in KT like economic/strategic outcomes and social outcomes. Interviewees from academia and interviewees from social partners named both economic/strategic and social outcomes whereas interviewees from economic partners only mentioned economic/strategic outcomes.

#### Expected Economic and Strategic Outcomes Influencing the Attitude

The majority of interviewees from academia indicated the following expected strategic outcomes to positively influence their attitude towards KT: a stable cooperation with a big company to gain good reputation, building a network for future jobs for PhD students and testing the robustness of the innovation in practice. All interviewees from social partners mentioned the following expected strategic outcomes to positively influence their attitude: Contribution to close practical gaps and a high relevance of the expected KT outcomes for care structures, professionals and patients in healthcare. Interviewees from economic partners only named economic and strategic outcomes to positively influence their attitude and mentioned them more than other interviewees. The majority of interviewees from economic partners reported that the KT outcome needs to be a concrete added value for the company such as creating a better product than competitors or increased production and sales. Divergent expected outcomes of academic, social and economic partners were observed. All interviewees are aware of these different expectations and are able to understand other and oppositional perspectives. One interviewee from academia stated:

*“The transfer worked out better when the outcome was directly apparent for the company and you had the feeling that a concrete added value emerged for the company [...]”* (I03)

One interviewee from an economic partner stated:

*“The University wants to gain great knowledge, publish and improve its reputation. The company has to make money efficiently and survive on the market in the long term.”* (I14).

The expected outcomes that overlapped in academic and practice partners are the exploration of new topics, preparation for upcoming technological development, and staying up to date. These have been reported to especially positively influence their attitude toward KT outcomes and thus their engagement in KT.

#### Expected Social Outcomes Influencing the Attitude

In general, social outcomes (e.g., social participation) were mentioned less than economic and strategic outcomes (e.g., higher return of investment). Interviewees from economic partners did not mention social outcomes at all. The majority of interviewees from academia mentioned that for their attitude to be positively influenced, the expected KT outcome should be meaningful for practice and should contribute to the development of practice. As one interviewee said:

*“My main interest is to contribute to the further development of practice.”* (I07)

This is in accordance with the social outcomes mentioned by the majority of interviewees from social partners. The majority of interviewees from social partners consider it as their responsibility towards their clients to engage in KT and to support them with the newest technology. Furthermore, two interviewees from social partners mentioned that what positively influenced their attitude towards KT had changed over time. This is pointed out by the following quote:

*“In the beginning we thought that our clients would have a direct benefit if we engaged in KT projects [...] meanwhile our expectations have changed, it had become clear that in many projects it is only about participating in a certain development step and the expectation is to be helpful. We have a responsibility towards our clients to engage in research and it is not about our benefit.”* (I12)

The statement above shows that the expected outcomes that positively influence the attitude towards KT can change over time. This is an interesting finding and supports the possibility to agree on an overlap of expected outcomes between academic partners and practice partners.

### Pressure to Transfer or Access Knowledge

The only pressure perceived by most interviewees from academia was economic pressure such as funding organizations require KT in research projects to make funding available. Interviewees from economic partners did not mention to perceive any pressure at all to engage in KT. None of the interviewees perceived organizational or institutional pressure as such. The majority mentioned intrinsic motivations in KT engagement or that the topic of KT had become more relevant and thus structures within the organization had changed accordingly.

#### Organizational Pressure

None of the interviewees from academia reported to perceive any organizational pressure to engage in KT. Instead, they reported intrinsic motivation to engage in KT, demonstrated by the following quote:

*“I think it is important that you yourself see and pursue this as a central objective.”* (I07)

Some interviewees from academia stated:

*“My impression is not that this was brought in from the outside, but it was the spirit that the people involved shared and the interest to participate in such a cluster.”* (I04).

The same was confirmed by the majority of interviewees from social partners, made clear by the following quote:

*“People have a high personal interest to participate in such research because they find it exciting, or they feel it is a great thing.”* (I11)

Interviewees from both economic and social partners reported to have a department within the organization which was either concerned with scouting new technology, or an administrative department for technical assistance systems and digital participation who helped structuring the KT process. However, these departments were not perceived to exercise pressure, but rather gave the topic more relevance and structure within the organizations.

#### Economic Pressure

Some interviewees from academia mentioned that KT is a prerequisite to receive funding in many calls for proposals. Moreover, interviewees from academia pointed out that in the calls for proposal the transfer into practice and the subsequent economic and scientific exploitations play an important role. There is a sense that economic partners engage in KT because they primarily need funding for their research and KT seems to be a secondary objective. Interviewees from economic partners did not mention any economic pressure to engage in KT. They stated that mostly they get approached by the research institutes to engage in KT.

#### Institutional Pressure

Overall, the majority of interviewees from academia and interviewees from practice partners did not perceive institutional pressure by policy making and regulating institutions. A few interviewees from academia reported that in the past years research funding both at EU and federal level focused more on how knowledge can be applied in practice. This was also said to be increasingly reflected in the calls for proposals and in the requirements that need to be fulfilled in the project proposals to receive funding.

### Control Over KT

When asked about capabilities as a condition for control over KT, mainly organizational capabilities were mentioned.

#### Organizational Capabilities Needed for Successful KT

When asked what is needed for a successful KT, four broad aspects emerged from the interviews which can be bundled under the heading of project management. Personnel management and communication management were emphasized in all the interviews. Furthermore, appointment and time management, and financial management were pointed out. All interviewees agreed that resource planning is a major issue in the KT process. What stands out is that all interviewees highlighted the importance of a project manager on both sides who continuously accompanies the project. They mentioned that this project manager needs to overtake tasks in several categories. The task categories mentioned most often were managing, coordinating and planning the setup of the KT project. It was reported that in many projects, joint project management was a challenge that jeopardized KT. Some of the interviewees reported that successful KT, among other things, mainly depends on whether the project manager had already worked on both sides (i.e., in academia and in practice). Problems with joint project management might be attributed to difficulties in balancing the project needs of management with the academic needs of generating scientific insight.

The project manager plays a central role in communication management. Communication factors were mentioned to be crucial for successful KT, yet often missing. Working conditions, structures. and expectations need to be harmonized through continuous, planned, open and clear communication. This is demonstrated by the following quote:

*“It has to be clarified who is responsible for what, who delivers what and who can actually do what. When this is clarified you know on which basis you can communicate and work in daily doing, which exchange formats you can generate and how you describe the status quo”* (I13).

Interestingly, both interviewees from academia and interviewees from practice partners mention the same issues and improvement points to enhance the communication. Additionally, a positive work atmosphere was mentioned to lead to better cooperation. Interviewees from social partners and economic partners repeatedly expressed the wish to work with academic partners on equal terms.

In order to achieve sustainable results, the whole KT process needs to be embedded in an initial phase prior to the grant application (the *pre-project phase*) and a phase when the KT project has ended (the *post-project phase*). The need for face-to-face meetings in the pre-project phase was named frequently. Additionally, time management issues when writing the grant proposal for a KT project in the pre-project phase were reported to be problematic. During the KT project, regular meetings and test phases were mentioned to be missing, but highly important. Moreover, in the post-project phase, a closing event to present innovations and discuss the cooperation were mentioned to be important for successful KT. Interestingly, some interviewees mentioned that timing when writing the grant proposal is important for successful KT, indicating the need for a joint development of strategic goals. The majority of interviewees from practice partners reported that they would have liked to be included more in the application writing phase. This is pointed out by the following quote of an interviewee from a social partner:

*“We need to be perceived more as full partner, often we get asked to sign something we have not been informed about or included in just 2 weeks before the submission of application. We want to be included in this process to understand the project goals and to see where we fit in as a partner.”* (I10).

Additionally, all interviewees expressed their wish for more regular joint meetings to realistically plan the cooperation, give status quo updates and define responsibilities. All interviewees but mainly interviewees from practice partners reported the wish for more time slots for test phases of the innovation in the practice environment. The insufficient planning of test phases in the practice environment was highlighted to threaten KT because needs and conditions of practice partners were not included in the innovation development process. Further, there were some suggestions from interviewees from both, academia and practice, to host a closing event for a KT project to reflect on the cooperation, show the innovation and talk to potential financial supporters. Remarkably, both, academia and practice, are aware of the importance of this factor yet have not made attempts to implement it.

Interestingly, the majority of interviewees from social partners stated that missing sustainable financing support after the KT process to be a great barrier for successful KT. This is demonstrated by the following quote:

*“Missing sustainable financial support is the biggest obstacle why innovations do not end up in social services, because we do not have the means to finance them.”* (I10).

The question raised repeatedly by social interviewees was: Who is responsible for sustainable financial support? One interviewee from a social partner pointed out.

*“There is the question of who actually pays for it: the health insurance, the nursing care insurance, the care facilities or the patients themselves”* (I09).

A few interviewees from social partners suggested the inclusion of a third partner in the KT project for the implementation phase following the KT project. Optimally, this third partner should collaborate with the social partners on a long-term basis. Interviewees from social partners mentioned that this third partner could be a start-up or a health insurance company. As an alternative to a third partner, they suggested new political regulations as a possible solution for missing financial support for social partners. One interviewee from a social partner stated:

*“This is exactly the crux of the matter because, for example, aids are paid for by the health insurance companies, but this means that the research result or product must also be recognized as an aid. This may take years of work before it is recognized and before it can find its way into a refinancing cycle.”* (I08).

Interviewees from economic partners did not mention financial management to be problematic.

#### Technological Capabilities Needed for Successful KT

When engaging in technological KT with research institutes the question arose whether the relatively low technological capabilities of the practice partners were believed to be a barrier to successful KT. Neither academic nor interviewees from practice partners believe that they need the same technological capabilities for a successful KT, only a common ground for communicating about technological subjects was thought necessary. However, the need for sustainable technological support after the KT project was mentioned by several interviewees from social partners and seems to be a major problem for sustainable KT for social partners. One solution proposed by interviewees from social partners was to include a third partner in the KT process such as a start-up that takes over the part of technological support and the development of an end product. Many interviewees from social partners repeated the issue of missing sustainable support:

*“We had prototypes, but not end products and that is where the difficulty starts. [...] The university has a mandate to do research and we are a classic user, [...] there is a third component missing and that is exactly this transfer component. How is such a research result transformed into a product and how can this product be refinanced to go into practice?”* (I08)

Another topic raised by the majority of interviewees from social partners was the wish for a more detailed initial analysis of the technical conditions at their site in the pre-project phase in order to ensure that the innovation can be implemented. Interviewees from economic partners did not raise such issues.

#### Institutional Capabilities Needed for Successful KT

KT processes are framed by numerous contextual conditions. Especially, interviewees from academia repeatedly emphasized the tight timeframe for the grant proposal writing as funding agencies only foresee a few weeks between publication of call and grant proposal deadline (e.g., the Bundesministerium für Bildung und Forschung (BMBF) in this field often foresees 2 months: German Federal Ministry of Education and Research, 2020a; German Federal Ministry of Education and Research, 2020b; German Federal Ministry of Education and Research, 2021). Interviewees from economic partners reported this to be problematic as well:

*“What has been missing in the projects so far is realistic work planning in the applications.”* (I14)

Furthermore, social recipients commented:

*“We have the opinion that this temporary project structure is not particularly useful.”* (I11)

In particular, interviewees from practice partners did not feel sufficiently included in the application writing phase in most KT projects and thus felt that their aims were not sufficiently represented.

## A Multi-Directional and Agile Transfer Strategy

**FIGURE 2:**
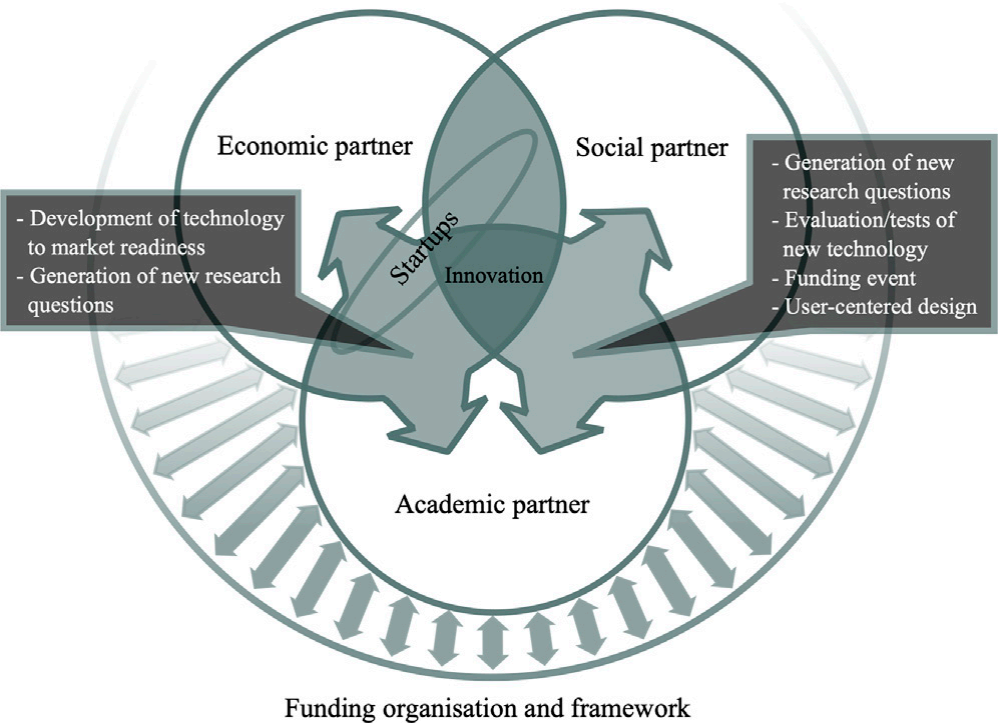
Multi-directionality of cross-sectoral collaboration.

In sum, the main aspects that kept recurring in our analysis as being essential for a successful KT strategy can be attributed to *multi-directionality* and *agility*. This means that all KT partners should participate at equal level so that they can continuously contribute their own insights into the KT process. This requires additional resources such as resources for a joint project manager. In practice, the equal partnership of collaboration partners is limited in current cross-sector projects as stated in the interviews and literature by Jansson et al., 2009. However, to achieve successful KT, ideally practice partners should take on an equal role and be more integrated in the whole KT process (*agility*), instead of being mere recipients. Additionally, a successful KT strategy is not limited to bilateral relationships but extends to third partners and funding bodies (*multi-directionality*, see Figure 2).

### Positive Attitudes Towards KT by Finding an Overlap of Expected Outcomes

In general, academic partners, social and economic partners expect different KT outcomes. Interestingly, in this study, the opposing parties are able to anticipate the other party’s expected outcomes. Differences in expected KT outcomes do not hamper successful KT per se but need to be explicitly addressed prior and during the KT project to ensure that they receive sufficient resources. Therefore, research institutes need to find an overlap between their own expected outcomes and those of certain practice partners. This overlap has to be clearly communicated between academic partners and practice partners. Thus, open and dynamic communication between the partners characterized by a communication style that builds trust, teamwork and consensus, also when not currently engaged in a KT together, can positively influence the attitude towards KT. As mentioned, before it also needs to be considered that outcome expectations can change over time.

Our findings on the attitude towards KT further support the idea of a highly agile KT strategy for research institutes. Therefore, we suggest the following measures for successful KT.

#### Communication

Communicate with practice partners clearly before and during the KT process to:

• Establish regular professional exchange (project manager task)
• Identify and formulate an overlap of the expected KT outcome and formulate diverging KT outcomes and resources.

### Organizational, Technological and Institutional Capabilities for Control Over KT

#### Organizational Capabilities

Personnel and communication management were mentioned to be the most influencing aspects for successful KT. For these two aspects the KT project manager should have the main responsibility. Ideally, the project manager has worked on both sides before (research and practice) and knows the work organizations and logics of both partners. Another solution proposed by Innovate United Kingdom (2020) is to engage in a knowledge transfer partnership. A knowledge transfer partnership is a partnership of a company, an academic research institute and a university graduate project lead. The graduate works as a project manager at the business and ensures communication flow between the other two actors (Innovate UK, 2020).

Our study revealed that a project manager should use the control over personnel to precisely plan human resources. Especially the allocation of personnel resources to project roles was highlighted to be crucial for successful KT (see measures below). To ensure that people are able to sufficiently contribute to the joint goals of the KT project continuous information flow is necessary. As mentioned in previous studies, the project manager has an important role as connector between organizations and has to guarantee effective communication (Loo, 2002).

A positive work atmosphere that allows for open and transparent communication was expressed from both sides to be crucial for joint decision-making. This corresponds with the literature on KT in successful partnering projects (Hudcova, 2014) and tools of internal communication in KT (University of Kansas, n. d.) and requires trust. Therefore, trust between academic partners and practice partners encourages their willingness to share knowledge (Hudcova, 2014; University of Kansas, 2021). Also, equal participation, as wished by all partners, can be ensured by a positive work atmosphere and a balanced dialogue (Hudcova, 2014).

Continuous communication organized by the KT project manager was equated to regular, planned meetings (appointment management), to update each other on the status quo and make coordinated decisions for further procedure in the project. Prior studies agreed that planned meetings are more valuable in terms of KT than unplanned meetings (Hudcova, 2014). This is the case because planned meetings should have a stated objective and people attending the meeting have to come prepared. Furthermore, the project manager needs to systematically structure the meeting to increase efficiency of the meetings (Hudcova 2014). This study supports the evidence from previous studies that the frequency of planned meetings in KT projects should be increased (Hudcova, 2014).

Besides the frequency of meetings also the group size and the circumstances under which people meet need to be considered. Face-to-face meetings of small groups or face-to-face meetings of two people were reported to be the best size according to (Hudcova, 2014). However, if face-to-face meetings are not possible because organizations are located at distant locations, a common work platform and the alignment of digital tools can enhance communication (Riege, 2005). Our study revealed that in KT projects often neither work platforms nor digital tools are aligned between the different partners. This is the case because the partners have different working processes and goals.

Furthermore, the content of the meetings of the KT project partners needs to be considered. Increased information exchange by feedback and status quo updates in these meetings will ensure for continuous communication. Immediate, quick and authentic feedback, after a task has been completed, allows for frequent adjustment of the work modes and work pace of the partners (Hudcova, 2014). Besides regular meetings, our interviewees considered appointment management aspects before, during, and at the end of the KT project necessary. Increasing practice partners’ involvement in the grant proposal writing phase will firstly increase the quality of the proposal and secondly prevent disappointment and frustration of the partners. When being left out of the writing process or only being included in the writing process on short notice, practice partners do not have the opportunity to bring in their own ideas and goals. In general, different tool kits for writing successful proposals and also the guidelines for outlining proposals from the BMBF do not mention the inclusion of the practice partner in the application writing phase to be essential (University of Kansas, n. d.; BMBF, 2017).

Moreover, the majority of practice partners and a few academic partners indicated that not only the appointment management in the grant proposal writing phase was important but also the appointment management during the KT projects needed improvement. Missing time resources for test phases in the practical environment (e.g., testing a developed system in the lab as well as in a clinic, care home or company during the KT process) were mentioned by both academic partners and practice partners. They highlighted that timed and repeated cycles of test phases are important for successful KT. In addition to the practice partners, the end users of the innovations should be involved more frequently in test phases. Their participation increases their commitment during KT and ensures that the innovation is tailored to their needs and work conditions. End users are more likely to use the innovation in daily life after the KT project. Interviewees suggested to plan test phases whenever a new feature of the innovation is available. In reviewing the literature, no data was found on the best time intervals to implement test phases. However, a model of Systematic Transfer of Knowledge from Mittelmann (2016) highlights the importance of test phases during the KT project. Additionally, both academic partners and practice partners mentioned that at the end of a KT project a closing event should take place to present the innovation to future financial supporters. This event should be used to discuss the lessons learned from the collaboration during the KT project and further steps. Therefore, we suggest the following measures.

##### Communication

Assign role of continuous project manager who:

- Connects organizations, plans human resources
- Ensures open, dynamic, targeted, effective, regular, structured communication, manages meetings and ensures regular feedback in face-to-face meetings (two people or small groups size)
- Enables communication *via* a common work platform and aligns digital tools
- Ensures commitment to and responsibility of tasks of staff
- Establishes positive work atmosphere that allows for open and transparent communication
- Has previously worked on both sides, academia and practice and is familiar with both work organizations logics and tasks

##### Test Phases

• Plan recurring test phases for practice partner and end user in practice environment when new feature of innovation is available

##### Closing Event

Performing Closing Event

- Present the innovation to future financial supporters of the innovation and discuss the lessons learned from the collaboration during the KT project and future plans.

##### Contact

Stay in contact with practice partners also when not currently engaged in KT project together so that:

• The practice partners are involved in the application writing phase from the beginning and time is used efficiently in the application phase.

#### Social Partners Missing Sustainable Financial and Technological Support

Interestingly, only social partners mentioned that missing sustainable technological support and financial support are a barrier to successful KT. They stated support to be missing in two phases: 1) in the development phase from prototype to end product and 2) in the phase when updates and new licenses of the innovation are employed. Both research institutes and social partners reported not to be required to develop and distribute technological end products. Thus, social partners proposed to include a third partner to take on these tasks in the implementation phase following the KT project. The interviewees from social partners reported that the third partner could be a start-up or a health insurance company that provides technological and financial support when the KT project ends. Fini et al., 2014 stated that more universities install Technology Transfer Offices that are conductive to spin-off creations and have increased the number of spin-offs from universities. However, the creation of spin-offs in a university context involves many actors and is a highly complex task (Fini et al., 2014). Therefore, academic partners should consider conditions and capabilities of potential social partners when choosing their partners for KT projects. We suggest the following measures for successful KT.

##### Financial and Technological Support for Social Partners

- Include third partners like health insurance or start-up from the beginning of KT project so they take over the part of sustainable financial and technological support.
- The role and responsibilities of the third partner need to be communicated clearly and transparently before collaboration begins.

#### Institutional Capabilities and General Conditions of Calls for Proposals

The framework conditions and standards of existing calls for proposals and their negative impact on KT project were expressed by both, academic and practice partners. On the one hand, the tight time window for application writing was mentioned. On the other hand, the high standards of funding agencies for the selection of proposals for funding were reported to be an obstacle for the KT process as high reject rates require additional resources for repeated proposal writing and adaptation to highly specific call restrictions. This can yield an unbalanced relation between desired and realistic outcomes which can lead to disappointment and frustration in the long term. Funding agencies need to reconsider their funding strategies by allowing more time for the development and specification of joint outcomes, providing fewer restrictions on calls to reduce complexity for this process and to reconsider the allocation of resources to research and KT. The research institutes could seek contact with the funding organization and generate impulses for changing these regulations including structural problems with regard to personnel resources that are missing during the KT project, as for example the KT project manager who would actually be necessary *before* the actual start of the KT project and until *after* the end of the project in order to prepare and secure the achieved results... These findings could be seen as an incentive to engage in an open dialogue with funding organizations by which these issues could be addressed and discussed. We suggest the following measures for successful KT.

##### Contact Funding Organization

- Seek contact with the funding organizations to incentivize regulation change with regard to application timeframe and application framework and address missing personnel resources.

## Conclusion

**FIGURE 3:**
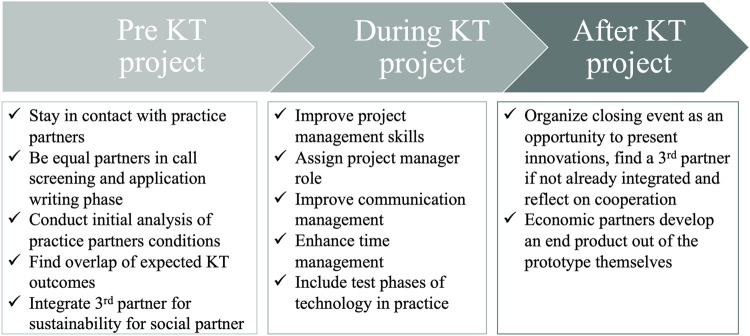
Three phases in an agile KT project.

In conclusion, research institutes need to adapt their KT strategy to the respective partners in an agile process (cf. *A Multi-Directional and Agile Transfer Strategy* Section) which includes phases prior to and after actual KT projects to ensure multi-directional KT through equal partnership (see Figure 3). The elements we found to be important for successful KT are in line with recent approaches to research and development as well as collaboration of departments of technology development and production in industry (Fraunhofer IAO, 2011, August, 09; Kienbaum, 2018, February, 19). The conditions and capabilities of the partners are crucial for successful KT and thus have to be considered in the KT strategy. In some cases, the research institute can only react to framework conditions (e.g., paradigm shift, standards of calls for proposals, mandate of practice partner). In many cases the research institute can actively steer and change its KT strategy (e.g., assign project manager role, include practice partner continuously), as it was elaborated in this study. It needs to be emphasized that realizing and applying the recommended measures for a successful KT strategy, requires intensive collaboration between the research institutes and the practice partner.

## Data Availability

The raw data supporting the conclusions of this article will be made available by the authors, without undue reservation.
