# Supplementary material for: A Multi-Directional and Agile Academic Knowledge Transfer Strategy for Healthcare Technology
Source: Front Robot AI. 2021 Dec 21;8:789827. doi: 10.3389/frobt.2021.789827 (PMC8724569; doi:10.3389/frobt.2021.789827)
Supplement: Supplementary file 1 [file Table1.DOCX]

Supplementary Material

# Interview guide for academic partners (providers) and practice partners (social and economic recipients)

**Introductory questions**

-What experiences have you had so far with the transfer of knowledge and innovation into practice?

-What expectations do you have when you participate in a project together with the academic partner?

**Inquiries:**

-Would you say that the knowledge transfer is successful in your case or is there still room for improvement?

-What do you hope for from a project together with the academic partner? e.g. prototypes, technical insights into new technical possibilities?

-Please be more specific, explain, could you give me an example

**Key questions**

**Question 1: Attitude:**

In your opinion, what are the advantages/ benefits of knowledge and innovation transfer to your practice partners?

**A. Inquiries:**

Would you also see a benefit for society in the generation and dissemination of knowledge - and why? (social outcome)

Do you assume that knowledge transfer will result in economic advantages for you / your institution / company - and why? (economic outcome)

Do you assume that knowledge transfer will result in strategic advantages for you / your institution - and why? (strategic outcome)

**B. Inquiries:**

Can you give me an example of this?

Can you further specify that?

Can you name any other advantages / benefits in other areas?

**Question 2: Pressure:**

Which people or institutions would like you to get involved in the transfer of knowledge and innovation to your practice partners**?**

**A. Inquiries:**

Does the university / research group / company expect you to get involved in knowledge and innovation transfer to your practice partners? (organisational pressure)

Do your funders expect you to get involved in the transfer of knowledge and innovation to your practice partners? (economic pressure)

Is your community (universities, science/research sector) expecting you to engage in knowledge and innovation transfer to your practice partners? (institutional pressure)

**B. Inquiries:**

Can you give me an example of this?

Can you further specify that?

Can you name any other institutions?

Are there any binding guidelines for the KT? (internal / external)

**Question 3: Control:**

What is needed or what must be taken into account for knowledge transfer to succeed? Success condition?

**A. Inquiries:**

Do certain process features have to be taken into account for KT to succeed? (Knowledge Management,

Management of KT process) (organisational capabilities)

Do certain technological capabilities have to be available for KT to succeed? (Technological expertise, language) (technological capabilities)

In your opinion, would secure financing be beneficial for a successful KT? Funding (institutional capabilities)

**B Inquiries:**

Can you give me an example of this?

Can you further specify that?

Can you name any other capabilities that are required?

**Question 4:**

In your opinion, do the factors XY you mentioned have an equal influence on knowledge transfer? Are they all equally important?

**Question 5:**

Are there any other factors that you can think of that we have not mentioned yet?
